# Combination therapy versus pharmacotherapy, endoscopic variceal ligation, or the transjugular intrahepatic portosystemic shunt alone in the secondary prevention of esophageal variceal bleeding: a meta-analysis of randomized controlled trials

**DOI:** 10.18632/oncotarget.18143

**Published:** 2017-05-24

**Authors:** Lu-Lu Lin, Shi-Ming Du, Yan Fu, Hui-Yun Gu, Lei Wang, Zhi-Yuan Jian, Xian-Feng Shen, Jie Luo, Chao Zhang

**Affiliations:** ^1^ Center for Evidence-Based Medicine and Clinical Research, Taihe Hospital, Hubei University of Medicine, Shiyan 442000, China; ^2^ Department of Pharmacy, Taihe Hospital, Hubei University of Medicine, Shiyan 442000, China; ^3^ Department of Hepatobiliary Surgery, Taihe Hospital, Hubei University of Medicine, Shiyan 442000, China; ^4^ Department of Gastroenterology, Taihe Hospital, Hubei University of Medicine, Shiyan 442000, China; ^5^ Administrative Offices, Taihe Hospital, Hubei University of Medicine, Shiyan 442000, China; ^6^ Hepatopancreatobiliary Surgery Treatment Center, Taihe Hospital, Hubei University of Medicine, Shiyan 442000, China

**Keywords:** esophageal varices, liver cirrhosis, endoscopic variceal ligation, transjugular intrahepatic portosystemic shunt

## Abstract

Patients with liver cirrhosis and variceal hemorrhage are at increased risk of rebleeding. We performed a meta-analysis toassess the clinical efficacy of combination therapy (pharmacotherapy and endoscopic variceal ligation (EVL)) compared with pharmacotherapy, EVL, or transjugular intrahepatic portosystemic shunt (TIPS) alone in the prevention of rebleeding and mortality. A literature search of MEDLINE, EMBASE, and the Cochrane Controlled Trials Register, up until November 2016, identified relevant randomized controlled trials. Data analysis was performed using Stata 12.0. Regarding overall mortality, combination therapy was as effective as EVL, pharmacotherapy, and TIPS (relative risk (RR) = 0.62, 95% confidence interval (CI): 0.36-1.08, RR=1.05, 95% CI: 0.68-1.63, and RR=1.39, 95% CI: 0.92-2.09, respectively). Combination therapy was as effective as EVL and pharmacotherapy alone in reducing blood-related mortality (RR=0.43, 95% CI: 0.15-1.25, and RR=0.42, 95% CI: 0.17-1.06), whereas TIPS was more effective than combination therapy (RR=5.66, 95% CI: 1.02-31.40). This was also the case for rebleeding; combination therapy was more effective than EVL and pharmacotherapy alone (RR=0.57, 95% CI: 0.41-0.79, and RR=0.65, 95% CI: 0.48-0.88), whereas TIPS was more effective than combination therapy (RR=9.42, 95% CI: 2.99-29.65). Finally, regarding rebleeding from esophageal varices, combination therapy was as effective as EVL alone (RR=0.59, 95% CI: 0.33-1.06) and was more effective than pharmacotherapy alone (RR=0.58, 95% CI: 0.40-0.85), although was less effective than TIPS (RR=2.20, 95% CI: 1.22-3.99). TIPS was recommended as the first choice of therapy in the secondary prevention of esophageal variceal bleeding.

## INTRODUCTION

Variceal rebleeding is a frequent and severe complication in cirrhotic patients. Patients who survive an episode of acute variceal hemorrhage are at increased risk of rebleeding and death. The median rebleeding rate in untreated individuals is approximately 60% and the mortality rate is 33% within 1-2 years of the hemorrhage [1–3]. Pharmacotherapy, endoscopic variceal ligation (EVL), and the transjugular intrahepatic portosystemic shunt (TIPS) are the recommended interventions for the prevention of variceal bleeding. Drug therapy, more specifically nonselective β-blockers or a combination of isosorbide mononitrate (ISMN) and nadolol, has been found to reduce portal pressure and prevent variceal rebleeding [4, 5]. Ligation is reported to be more effective at reducing patient mortality than sclerotherapy [6]. In addition, EVL achieves variceal obliteration with fewer endoscopic sessions and has been found to be effective in controlling active variceal bleeding [7–10]. The TIPS procedure is a minimally invasive, image-guided intervention used for secondary prevention of bleeding and as salvage therapy in acute bleeding [11]. TIPS were created with Wallstents (Schneider, Inc., Plymouth, Minnesota) using standard techniques described elsewhere, and effectively control bleeding in patients with refractory variceal hemorrhage [12, 13].

Several randomized controlled trials (RCTs) have reported the differences in efficacy between these interventions in the control of esophageal variceal bleeding. Although drug therapy was stated to be as effective as EVL in current studies [14, 15], it has also been reported that combination therapy is more effective than EVL or drug therapy alone for reducing the risk of rebleeding, although the effect on mortality was unclear [15, 16]. Moreover, there is evidence to suggest that TIPS is more effective at reducing rebleeding than drug therapy or EVL [17–19]. However, whether the TIPS is more effective than combination therapy (pharmacotherapy and EVL) has not been investigated. Therefore, we performed a meta-analysis of randomized trials to assess the efficacy of combination therapy (pharmacotherapy and EVL) compared with pharmacotherapy, EVL, or TIPS alone in the prevention of rebleeding and mortality in this study.

## RESULTS

### Characteristics of individual studies

**Figure 1:**
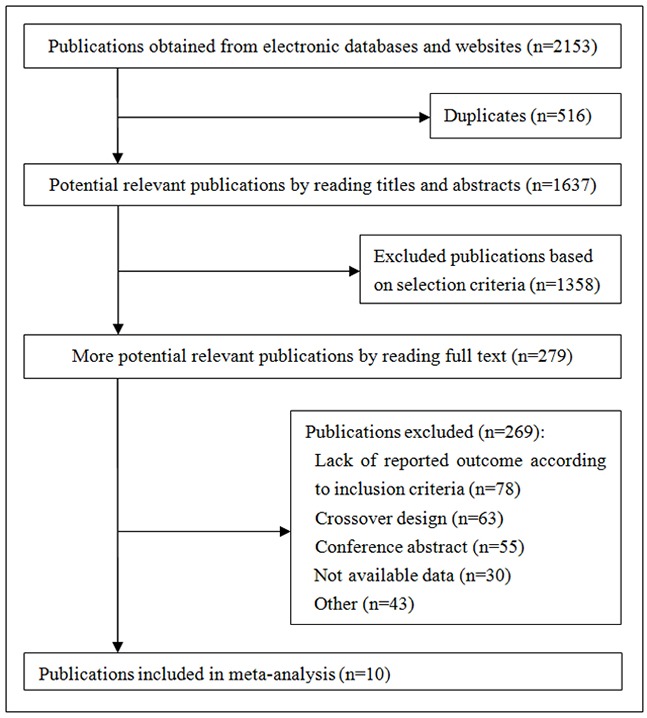
Summary of trial identification and selection

We identified 2153 publications from the electronic databases (Figure 1), of which 516 were excluded as duplicates and 1358 were excluded based on selection criteria. This resulted in 279 articles, which were independently read by two authors. Eventually, ten studies involving 1076 patients were included in our meta-analysis [20–29]. The characteristics of each individual study are presented in Table 1.

**Table 1:** The characteristics of each individual study

Study	Year	Country	Patients (C/M)	Mean age (C/M)	Male/female	Alcohol (C/M)	Viral (C/M)	Child score (C/M)			Combination therapy	Monotherapy	Follow-up time (months)
								A	B	C			
Lo [26]	2000	China	60/62	53± 11/51± 12	94/28	17/20	41/41	11/12	12/18	19/22	Nadolol 60mg/day, 1-2 bands	1-2 bands	22/21
De la Pena [27]	2005	Spain	43/37	60/60	60/20	27/26	112/8	6/6	25/20	2/11	Nadolol 58mg/day, EVL	NA	17.5/17
Jain [28]	2006	American	61/67	NA	NA	NA	NA	26/24	23/37	12/6	Propranolol 114.3mg/day, EVL	NA	NA
Kumar [29]	2009	India	88/89	42(14)/41(14)	153/24	33/30	13/20	35/26	31/34	10/15	Propranolol 120mg/day, ISMN 40mg/day, 2-10 bands	2-10 bands	15/15
Lo (a) [30]	2009	Taiwan	47/46	52±11/50±12	77/16	15/17	28/26	13/14	20/25	14/7	Terlipressin 4mg/day, 4 bands	Terlipressin 1 mg/6h for 5 days	NA
Lo (b) [31]	2009	Taiwan	60/60	54±10/52±11	87/33	21/15	31/40	20/21	29/31	11/8	Nadolol 40 mg/day, ISMN 20mg/day, EVL	Nadolol 40 mg/day, ISMN 20mg/day	22.3/22.7
Garcia-Pagan [32]	2009	Spain	80/78	57±12/56±11	118/40	39/42	25/18	16/18	46/42	18/18	Nadolol 36mg/day, ISMN 36mg/day, EVL	Nadolol 36mg/day, ISMN 36mg/day	14.4/15.3
Garcia-Pagan [35]	2010	Spain	31/32	49±6/52±10	44/19	20/22	5/4	NA	16/16	15/16	Propranolol 55mg/day, ISMN 25mg/day, EVL	e-PTFE–covered stents: 10mm	14
Luo [33]	2015	China	36/37	50.78±13.61/ 49.53±14.02	43/30	2/4	30/26	NA	25/24	12/12	Propranolol, 65.4 mg/day, 4-6 bands	e-PTFE–covered stents: 10mm	20.9/22.8
Holster [34]	2016	Netherlands	35/37	54/56	41/31	18/13	1/7	13/13	18/19	4/5	Terlipressin 6-12mg/day, 4.3 bands	Balloon-expandable stent: 8 mm in 21 patients, 10 mm in 10 patients	23

C/M, combination therapy/monotherapy; EVL, endoscopic variceal ligation; ISMN, isosorbide mononitrate; e-PTFE, extended polytetrafluoroethylene; NA, not available.

### Quality of the included studies

The risk of bias in the included studies was strictly evaluated. Details of methodological approach are presented in Table 2.

**Table 2:** The risk of bias in the included studies

Study	Year	Random sequence generation	Allocation concealment	Blinding of participants and personnel	Blinding of outcome assessment	Incomplete outcome data	Selective reporting	Other bias
Lo [26]	2000	Low risk	Low risk	Unclear	Unclear	Low risk	Low risk	Low risk
De la Pena [27]	2005	Low risk	Low risk	Unclear	Unclear	Low risk	Low risk	Low risk
Jain [28]	2006	Low risk	Low risk	Unclear	Unclear	Low risk	Low risk	Low risk
Kumar [29]	2009	Low risk	Low risk	Unclear	Unclear	Low risk	Low risk	High risk
Lo (a) [30]	2009	Low risk	Low risk	Unclear	Unclear	Low risk	Low risk	Low risk
Lo (b) [31]	2009	Low risk	Low risk	Unclear	Low risk	Low risk	Low risk	Low risk
Garcia-Pagan [32]	2009	Low risk	Low risk	Unclear	Unclear	Low risk	Low risk	Low risk
Garcia-Pagan [35]	2010	Low risk	Low risk	Unclear	Unclear	Low risk	Low risk	Low risk
Luo [33]	2015	Low risk	Unclear	Unclear	Unclear	Low risk	Low risk	Unclear
Holster [34]	2016	Low risk	Low risk	Unclear	Low risk	Low risk	Low risk	Unclear

### Overall mortality

**Figure 2:**
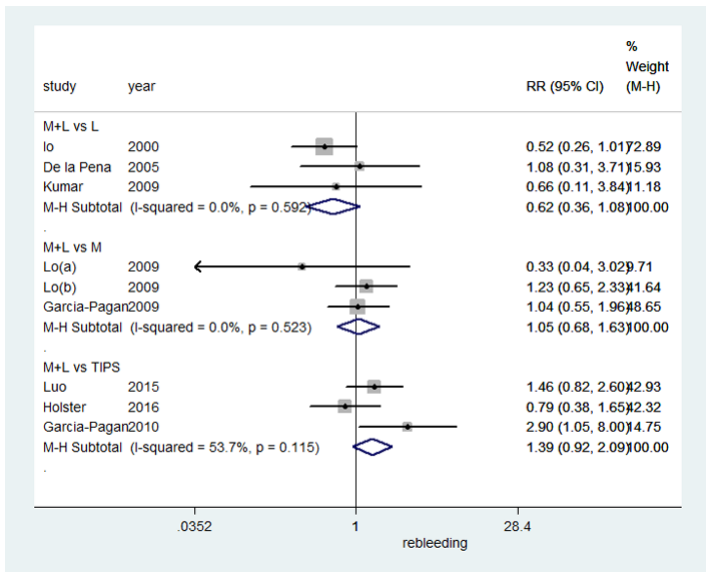
Forest plot of overall mortality

When assessing the effect on overall mortality (as shown in Figure 2), combination therapy did not significantly differ from EVL alone in a fixed-effects model (RR=0.62, 95%CI: 0.36-1.08, I^2^=0.0%, P=0.592). Compared to pharmacotherapy alone, combination therapy also had no significant effect on overall mortality in a fixed-effects model (RR=1.05, 95%CI: 0.68-1.63, I^2^=0.0%, P=0.523). Similarly, combination therapy versus TIPS did not show a statistically significant difference in a random-effects model (RR=1.39, 95%CI: 0.92-2.09, I^2^=53.7%, P=0.115).

### Mortality caused by variceal bleeding

**Figure 3:**
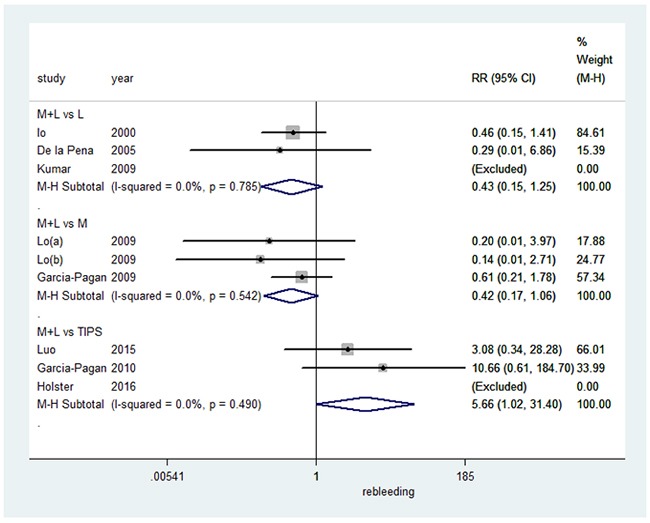
Forest plot of mortality caused by variceal bleeding

We also assessed the effect on mortality caused by variceal bleeding (summarized in Figure 3), and found that combination therapy did not significantly differ from EVL alone in a fixed-effects model (RR=0.43, 95%CI: 0.15-1.25, I^2^=0.0%, P=0.785). Compared to pharmacotherapy alone, combination therapy also had no significant effect on mortality caused by variceal bleedingin a fixed-effects model (RR=0.42, 95%CI: 0.17-1.06, I^2^=0.0%, P=0.542). However, TIPS resulted in a significant decrease in mortality caused by variceal bleeding when compared to combination therapy in a fixed-effects model (RR=5.66, 95%CI: 1.02-31.40, I^2^=0.0%, P=0.490).

### Recurrence of bleeding

**Figure 4:**
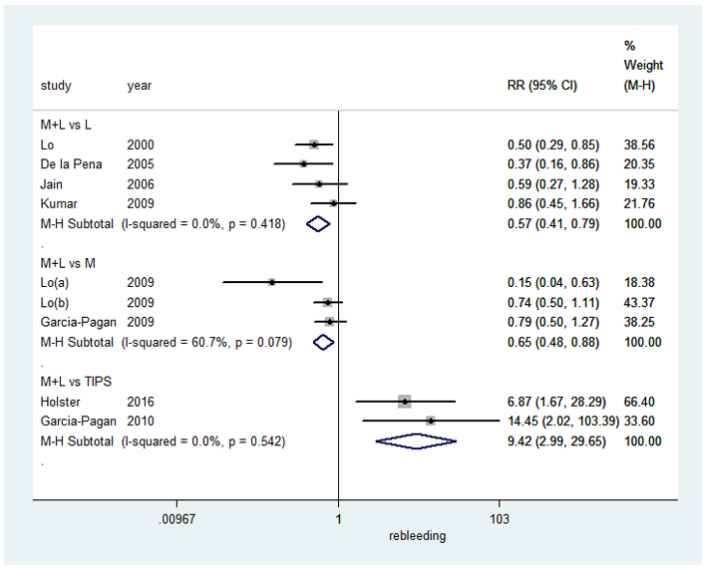
Forest plot of rebleeding

We next evaluated the effect on recurrence of bleeding (summarized in Figure 4). Combination therapy resulted in a significant decrease in the recurrence of bleeding (RR=0.57, 95%CI: 0.41-0.79, I^2^=0.0%, P=0.418), when compared to EVL alone in a fixed-effects model. In comparison to pharmacotherapy alone, combination therapy also significantly decreased the recurrence of bleeding in a random-effects model (RR=0.65, 95%CI: 0.48-0.88, I^2^=60.7%, P=0.079). However, TIPS significantly decreased bleeding recurrence when compared to combination therapy in a fixed-effects model (RR=9.42, 95%CI: 2.99-29.65, I^2^=0.0%, P=0.542).

### Recurrence of bleeding from esophageal varices

**Figure 5:**
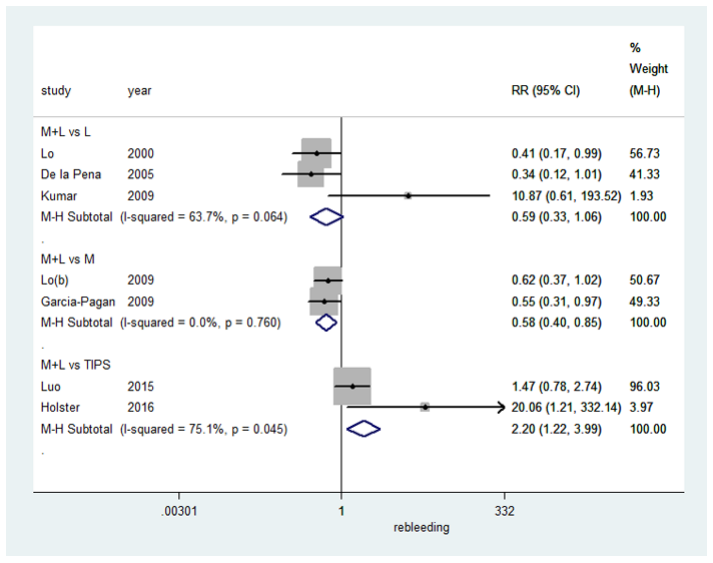
Forest plot of rebleeding from esophageal varices

Finally, we assessed the effect on recurrence of bleeding from esophageal varices (summarized in Figure 5). Combination therapy did not significantly differ from EVL alone in a random-effects model (RR=0.59, 95%CI: 0.33-1.06, I^2^=63.7%, P=0.064). Compared to pharmacotherapy alone, combination therapy resulted in a significant decrease in the recurrence of bleeding from esophageal varices in a fixed-effects model (RR=0.58, 95%CI: 0.40-0.85, I^2^=0.0%, P=0.760). However, TIPS significantly decreased esophageal bleeding recurrence in comparison to combination therapy in a random-effects model (RR=2.20, 95%CI: 1.22-3.99, I^2^=75.1%, P=0.045).

### Publication bias

No publication bias was observed for any of the outcomes based on the symmetry of the funnel plots, as shown in Supplementary Figures 1–4. The results of the Egger's test indicated no significant difference in any of the outcomes: overall mortality (Bias=0.514, 95%CI: -3.291-2.263, P=0.675), blood-related mortality (Bias=0.339, 95% CI: -2.393-3.072, P=0.763), recurrence of bleeding (Bias=1.280, 95%CI: -2.257-4.816, P=0.421), and recurrence of bleeding from esophageal varices (Bias=1.724, 95%CI: -1.785-5.233, P=0.262).

## DISCUSSION

Despite the administration of vasoactive drugs [30, 31], ligation of varices often combined with drugs [14], or placement of a TIPS, there is still a 15% to 20% mortality rate within 30 days, and an increased risk of rebleeding (up to 25%) within six weeks [32, 33]. Currently in the clinic, most studies have revealed that EVL is an appropriate substitute for endoscopic sclerotheapy, as EVL achieves variceal obliteration quicker, thereby resulting in lower rebleeding rates [16, 34, 35]. The combination of pharmacotherapy and EVL could be more effective than monotherapy (pharmacotherapy and EVL alone). In addition, TIPS is a new therapeutic modality for variceal bleeding with recognized results [36, 37]. Accordingly, this study evaluated the clinical benefit of pharmacotherapy plus EVL compared with pharmacotherapy and EVL alone, and TIPS.

This meta-analysis evaluated the clinical benefit of combination therapy, pharmacotherapy and EVL, compared with that of pharmacotherapy and EVL alone. Combination therapy was found to be more effective than monotherapy (pharmacotherapy and EVL alone) at preventing rebleeding, which was consistent with other studies [15, 38, 39]. The rationale for combining drug therapy with EVL is that they act through different mechanisms; EVL reduces variceal size, and drug therapy lowers portal pressure [20]. However, when assessing rebleeding from esophageal varices, the benefit of combination therapy was only observed in comparison to pharmacotherapy alone, and not with EVL. This could be attributed to the number of patients and events in analysis of the two subgroups. There was no statistically significant difference between combination therapy and monotherapy in the all-course mortality rate or mortality caused by bleeding. Similar findings have been reported by Thiele et al., who suggested that the combination of EVL and medical therapy could reduce the risk of rebleeding, but not overall mortality [38]. Gonzalez et al. provided conflicting evidence, suggesting that combiantion therapy can reduce the risk of mortality [39]. This difference in findings could be attributed to the sample size.

We provide evidence that TIPS was superior to combination therapy in reducing the risk of rebleeding and rebleeding from varices, in our meta-analysis. This is consistent with previous studies where TIPS was found to be more effective in preventing recurrent esophageal variceal bleeding in patients [32, 33, 34]. TIPS involves establishing a direct pathway between the hepatic and portal veins to decompress portal venous hypertension, which is the source of the patient's bleeding. Accordingly, TIPS is more than 90% effective in controlling bleeding from gastro-esophageal varices [11]. In our study, TIPS reduced mortality caused by bleeding, however, overall mortality was not significantly altered when compared to combination therapy. Holster et al. reported results consistent with this study [33], whereas a study by Garcia-Pagan et al. suggested a decrease in the risk of mortality with TIPS [29]. These contrasting results could be related to the patient follow-up or to the differing grades of cirrhosis inpatients. Sauer et al. demonstrated that TIPS did not improve survival rate associated with an increased risk of encephalopathy and high rates of shunt dysfunction [40]. There is an increase in the rate of development of hepatic encephalopathy after a TIPS procedure [13, 40, 41]. Conversely, other studies indicated that TIPS did not significantly increase the incidence of hepatic encephalopathy, compared other interventions [18, 27, 29, 37]. Although there is no consensus in these studies, TIPS is a widely accepted therapy as a result of extensive clinical validation in recent years. Based on Puente's study [42], we found that confounding factors including the Child score (Child C >20% and Child C ≤20%) and follow-up time (<15 months and≥15 months) weren't discovered to influence the results under in the case of the less number.

To our knowledge, TIPS insertion leads to important pathophysiologic circulatory changes; TIPS significantly reduces pressure in the extrahepatic portal venous system secondary to a dramatic drop in intrahepatic vascular resistance to portal flow; therefore, TIPS is potentially useful for patients with portal hypertension [43]. TIPS prevents rebleeding more effectively than drug treatment or endoscopic procedures alone, but it can cause encephalopathy and has no overall survival benefit [44]. Moreover, high-risk patients (those with advanced cirrhosis) experience less rebleeding and have an increased survival rate if TIPS is placed within five days of variceal bleeding [29, 44]. In this meta-analysis, we compare TIPS with combination therapy and show a significant reduction in mortality from variceal bleeding, although there was no overall improvement in survival when TIPS was used, which may be related to hepatic encephalopathy.

An advantage of this meta-analysis is that all included studies were randomized, controlled clinical trials and with large sample sizes [21], however, some limitations in our study should be addressed. Firstly, few clinical trials met the inclusion criteria, therefore, more clinical studies are required to confirm our results. Secondly, the double-blind methods of methodological quality of eligible trials could not be performed, due to the specificity of EVL and TIPS. In addition, heterogeneity of drug dose may also be a concern in our meta-analysis. Finally, the complications associated with TIPS, such as hepatic encephalopathy, are unclear in our study, which may exert influence on mortality.

## MATERIALS AND METHODS

### Literature search strategy

This systematic review and meta-analysis was reported according to Preferred Reporting Items for Systematic Reviews and Meta-Analyses guidelines [45] and conducted in accordance with the Cochrane Collaboration's systematic review framework [46]. We used the PubMed, EMBASE, and Cochrane Central databases to perform a literature search on articles published up until November 2016, using the following MeSH words and key terms: “esophageal varices”, “variceal rebleeding”, “variceal hemorrhage”, “portal hypertension”, “liver cirrhosis”, “pharmacotherapy”, “endoscopic variceal ligation”, and “transjugular intrahepatic portosystemicshunt”. We also searched the reference lists of the retrieved studies.

### Literature selection and exclusion

The inclusion criteria for selection of clinical trials in to the meta-analysis were as follows: (1) randomized, controlled trials comparing pharmacotherapy plus EVL with EVL or pharmacotherapy alone, or TIPS; (2) study participants should be older than 16 years of age with at least one previous episode of esophageal bleeding; and (3) studies needed to have measured at least one of the following outcomes as their endpoints: overall mortality, mortality caused by variceal bleeding, recurrence of bleeding, or recurrence of bleeding from esophageal varices.

Studies comparing these outcomes in the primary prevention of gastroesophageal bleeding, those that included patients with gastric varices alone, or liver cancer, were excluded from our analysis. If that the study was a duplicate or study's data could not be extracted or obtained through contact with the author, were excluded.

### Data extraction

Data was extracted directly from the selected studies by two independent reviewers. In the case of disagreement, a third reviewer was consulted. The relevant information included study design, patient characteristics, interventions, controls, and four outcomes: overall mortality, mortality caused by variceal bleeding, recurrence of bleeding, and recurrence of bleeding from esophageal varices.

### Quality assessment of included studies

Two investigators independently evaluated the methodological quality of eligible trials using the Cochrane collaboration tool for assessing risk of bias [47] (random sequence generation, allocation concealment, blinding of participants and personnel, blinding of outcome assessment, incomplete outcome data, selective reporting and other sources of bias).

### Statistical analysis

This meta-analysis was performed using Stata 12.0. Dichotomous outcomes were expressed as relative risk (RR) with a 95% confidence interval (CI) [46, 48]. Heterogeneity between studies was also analyzed using chi-square tests, with the significance level set to P <0.1 [49]. No heterogeneity is observed when I^2^ =0%. However, when I^2^ >50%, studies were considered to have significant heterogeneity and a random-effects model was used to conduct the meta-analysis, whereas when I^2^ <50%, a fixed-effects model was used instead [46].

The symmetry of a funnel plot was used to qualitatively determine whether there was publication bias [50]. In the funnel plot, larger studies that provide a more precise estimate of an interventions effect from the spout of the funnel, whereas smaller studies with less precision form the cone end of the funnel. Asymmetry in the funnel plot indicates potential publication bias, which is assessed by the Egger's test for a quantitative detection of bias [51].

### Ethical approval

Not required.

## CONCLUSIONS

This meta-analysis indicated that a combined therapy of pharmacotherapy plus EVL was more effective in decreasing rebleeding than monotherapy. Furthermore, TIPS was superior to combined therapy in decreasing the risk of rebleeding, rebleeding from varices, and mortality caused by bleeding, although not overall mortality. Accordingly, we recommend TIPS for the prevention of variceal rebleeding in patients with cirrhosis.

## SUPPLEMENTARY MATERIALS FIGURES AND TABLES



## Abbreviations

EVB: esophageal varices bleeding
EVL: endoscopic variceal ligation
TIPS: transjugular intrahepatic portosystemic shunt
ISMN: isosorbide mononitrate
RCT: randomized controlled trials
RR: relative risk
CI: confidence interval
