# Supplementary material for: Combination therapy versus pharmacotherapy, endoscopic variceal ligation, or the transjugular intrahepatic portosystemic shunt alone in the secondary prevention of esophageal variceal bleeding: a meta-analysis of randomized controlled trials
Source: Oncotarget. 2017 May 24;8(34):57399–408. doi: 10.18632/oncotarget.18143 (PMC5593651; doi:10.18632/oncotarget.18143)
Supplement: Supplementary file 1 [file oncotarget-08-57399-s001.pdf]

Combination therapy versus pharmacotherapy, endoscopic variceal ligation, or the transjugular intrahepatic portosystemic shunt alone in the secondary prevention of esophageal variceal bleeding: a meta-analysis of randomized controlled trials

SUPPLEMENTARY MATERIALS

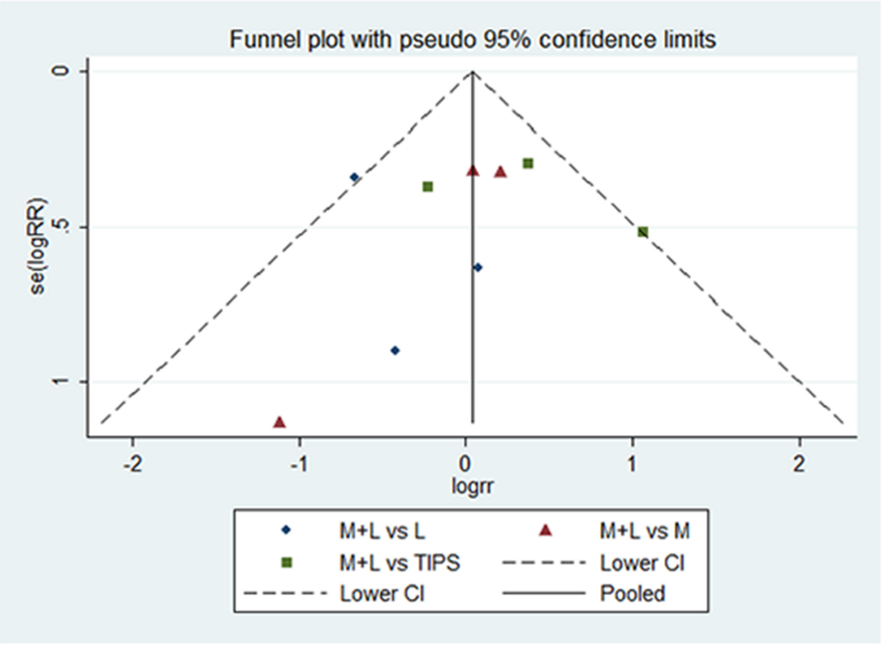

Supplementary Figure 1: Funnel plot of overall mortality.

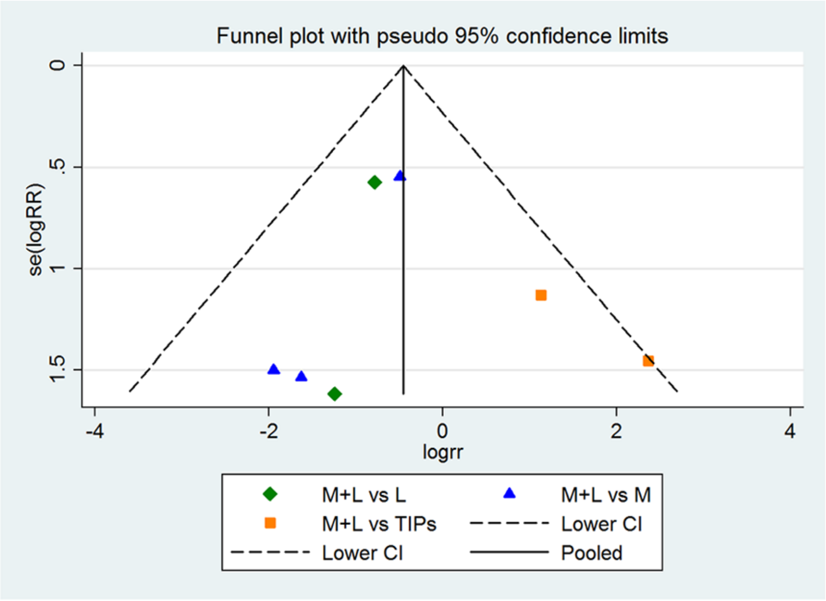

Supplementary Figure 2: Funnel plot of mortality caused by variceal bleeding.

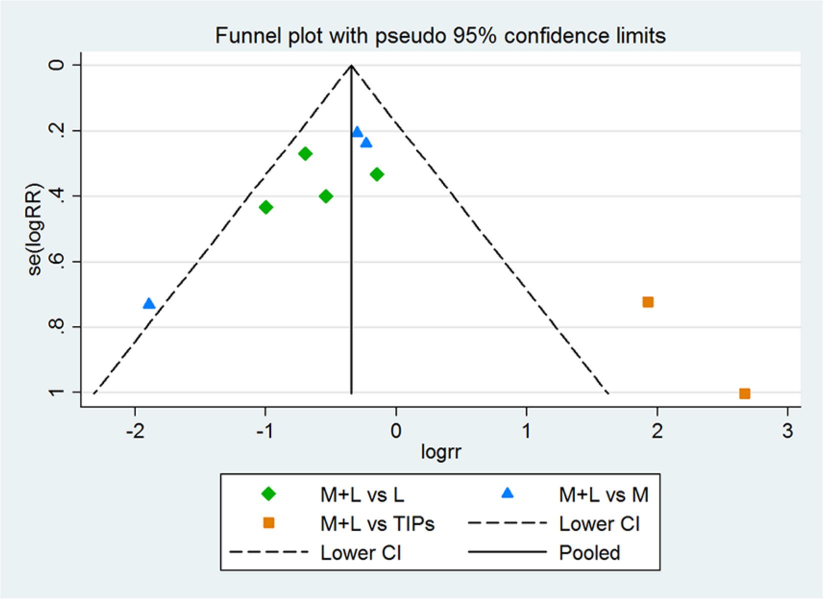

Supplementary Figure 3: Funnel plot of rebleeding.

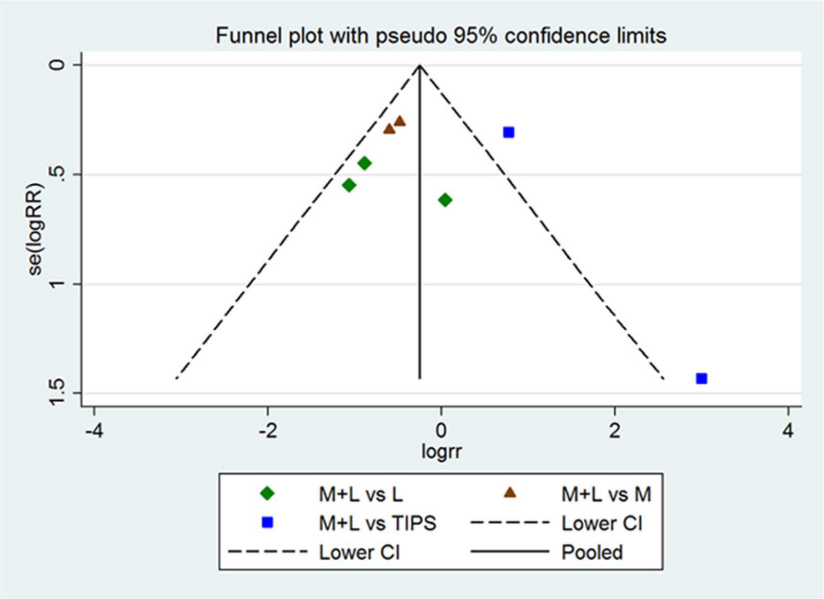

Supplementary Figure 4: Funnel plot of rebleeding from esophageal varices.
